# The relationship of women’s status and empowerment with skilled birth attendant use in Senegal and Tanzania

**DOI:** 10.1186/s12884-015-0591-3

**Published:** 2015-07-24

**Authors:** Kyoko Shimamoto, Jessica D. Gipson

**Affiliations:** Fielding School of Public Health, Center for Health Sciences, University of California, Los Angeles, 650 Charles E. Young Dr. South, 16-035, Los Angeles, CA 90095-1772 USA

**Keywords:** Maternal mortality, Delivery, Skilled birth attendant, Women’s status, Women’s empowerment, Cross-national study, Sub-Saharan Africa

## Abstract

**Background:**

Maternal mortality remains unacceptably high in sub-Saharan Africa with 179,000 deaths occurring each year, accounting for 2-thirds of maternal deaths worldwide. Progress in reducing maternal deaths and increasing Skilled Birth Attendant (SBA) use at childbirth has stagnated in Africa. Although several studies demonstrate the important influences of women’s status and empowerment on SBA use, this evidence is limited, particularly in Africa. Furthermore, few studies empirically test the operationalization of women’s empowerment and incorporate multidimensional measures to represent the potentially disparate influence of women’s status and empowerment on SBA use across settings.

**Methods:**

This study examined the relationship of women’s status and empowerment with SBA use in two African countries – Senegal and Tanzania – using the 2010 Demographic and Health Surveys (weighted births *n* = 10,688 in SN; 6748 in TZ). Factor analysis was first conducted to identify the structure and multiple dimensions of empowerment. Then, a multivariate regression analysis was conducted to examine associations between these empowerment dimensions and SBA use.

**Results:**

Overall, women’s status and empowerment were positively related to SBA use. Some sociodemographic characteristics showed similar effects across countries (e.g., age, wealth, residence, marital relationship, parity); however, women’s status and empowerment influence SBA use differently by setting. Namely, women’s education directly and positively influenced SBA use in Tanzania, but not in Senegal. Further, each of the dimensions of empowerment influenced SBA use in disparate ways. In Tanzania women’s higher household decision-making power and employment were related to SBA use, while in Senegal more progressive perceptions of gender norms and older age at first marriage were related to SBA use.

**Conclusions:**

This study provides evidence of the disparate influences of women’s status and empowerment on SBA use across settings. Results indicate that efforts to increase SBA use and to reduce maternal mortality through the improvement of women’s status and empowerment should focus both on improving girls’ education and delaying marriage, as well as transforming gender norms and decision-making power. However, given the multi-dimensional and contextual nature of women’s status and empowerment, it is critical to identify key drivers to increase SBA use in a given setting for contextually tailored policy and programming.

## Background

Maternal mortality is considered to be one of the greatest public health disparities of our time, as 99 % of maternal deaths occur in low-and middle-income countries and the vast majority of these deaths are preventable. This disparity is particularly pronounced in sub-Saharan Africa where the lifetime risk of maternal death is 1 in 38 women, as compared to the global average of 1 in 190 women [1]. Despite substantial reductions in maternal mortality in other regions, these reductions have been more limited in sub-Saharan Africa where the estimated Maternal Mortality Ratio (MMR) remains at 510 maternal deaths per 100,000 live births – more than twice as high as the global average of 210 [1].

Evidence indicates that survival for women and newborns improves with professional care at childbirth, such as that provided by a Skilled Birth Attendant (SBA). An SBA is defined as an accredited health professional – such as a midwife, doctor, or nurse – who has been trained to proficiency in the skills needed to manage normal (uncomplicated) pregnancies, childbirth and the identification, management and referral of complications in women and newborns [2]. Estimates indicate that with use of an SBA at all deliveries, 16–33 % of all maternal deaths could be averted [3].

Yet, despite the benefits of SBA, only half of deliveries are attended by SBAs in sub-Saharan Africa, and there has been little progress in increasing the proportion of SBA use over the past few decades [4]. A complex set of factors influence the likelihood of delivery care use and maternal mortality in low- and middle-income countries, including education and economic status, physical distance to facilities, availability of transportation, quality of care, and sociocultural norms/beliefs [5, 6]. Additionally, the status of women in their households and communities, as well as women’s power in deciding the type of care and provider is strongly predictive of delivery care use [5, 6]. The most recent articles also highlight the importance of women’s status and empowerment as one of the most critical factors for maternal health and attainment of other global health development goals [7, 8].

Women’s status and empowerment are terms that are commonly used to describe the social position of women and their ability to make decisions and to take action on issues affecting their well-being [9, 10]. In general, *women’s status* is defined as “women’s overall position in the society” [11], which encompasses their educational, cultural, economic, legal, and political position in a given society [6]. Women’s educational attainment is the most often used proxy measure of women’s status.

In contrast, *women’s empowerment* has been defined as the *process* by which those who have been denied the ability to make strategic life choices acquire such ability, comprising three inter-related dimensions – resources (as pre-conditions), agency (as process), and achievements (as outcomes) [9]. Women’s empowerment has been mostly operationalized and measured using proxy measures: women’s participation in household decision-making; access to, or control over household resources (e.g., income); perceptions of gender norms regarding the relationship between couples; and perceived equity in their power and resources [10, 12]. Early marriage and/or childbearing, as major “strategic life choices”, are also considered to be indications of women’s status and/or empowerment in a particular setting [13–16], in that they reflect broader gender norms regarding girls’ and women’s roles in society and the extent to which women are able to choose the occurrence and timing of these events with respect to other life aspirations [9, 17].

Previous examinations generally indicate positive relationships between women’s education and reproductive health outcomes, including delivery care use [18–30]. The effects of other sociodemographic characteristics, however, do not yield clear patterns across countries, or are less studied. For example, the effect of employment on delivery care use is mixed across African countries – positively in Ethiopia, Eritrea, Liberia, Nigeria and Mali, and negatively in Rwanda and Uganda [23–26, 29, 30]. Other potentially important influences such as marital and household relationship (e.g., polygamous/monogamous union; household headship), son preference, and the effects of age at first marriage and/or childbearing are rarely studied and have produced mixed results [16, 19].

Although many of the previous studies on delivery care use in Africa do not explicitly examine the effect of women’s empowerment [18–21], those that do generally find positive associations with delivery care use, yet the results are mixed across countries and regions in Africa [22–25]. For example, a meta-analysis found that household decision-making participation was positively associated with SBA use in 31 countries (including 21 African countries) [22]. Yet another multi-country study from Africa found that household decision-making was positively associated with facility delivery only in Nigeria, but not in seven other African countries [23]. A subsequent multi-level analysis of these same countries, however, found no significant effect between decision-making and facility delivery when accounting for clustering of countries [28].

In addition to a limited set of studies, examinations of the effects of women’s status and empowerment on delivery care use are further constrained due to the limitations of methodologies and differences in the ways in which women’s status and empowerment are conceptualized, operationalized, and measured across studies [10, 12]. For example, despite recognition of the complex, multidimensional, and culturally-defined nature and influence of empowerment on reproductive health, only a few studies consider the multidimensional structure of empowerment dimensions in a given setting [14, 24, 31–33], or examine the varied relationships between each measure of women’s empowerment and reproductive health behaviors [16, 23, 25, 34]. Only a few DHS studies on delivery care use examined both the multidimensionality and contextual differences in women’s empowerment in Africa by assessing the influence of various measures of empowerment across multiple countries [23, 25, 28, 29]. Furthermore, despite potentially synergistic effects of women’s empowerment and socioeconomic status on health outcomes, only one known African study has examined this interaction, finding significant effects between women’s autonomy by household wealth in predicting delivery care use [35].

To address the need for multidimensional and contextual examinations of women’s empowerment and its influence on SBA use in Africa, this study examined these relationships in Senegal and Tanzania, settings with similarly high levels of maternal mortality, yet with distinct sociocultural contexts. The aims are to first explore the structure and dimensions of women’s empowerment in these two settings, then to examine the effects of these constructs on SBA use in both countries.

## Methods

### Study settings

This study investigated the use of SBAs in Senegal (SN) and Tanzania (TZ). These two countries are similar with respect to maternal and child heath indicators, yet are culturally and economically different from one another. Infant and maternal mortality are similar across the two countries (50 per 1000 births in SN and TZ, and 320 per 100,000 live births in SN and 410 in TZ, respectively) [1], as are Total Fertility Rates (5.0 in SN and 5.4 in TZ) [36, 37].

At the same time, there are differences across the two settings with respect to income, health service use and availability, as well as sociocultural contexts. The national Gross Domestic Product per capita (GDP in USD) in Senegal (1032.7) is twice that of Tanzania (516.2) [38]. In Tanzania, half of the recent births in the last five years occurred at health facilities (50.2 %), compared to almost three quarters (72.8 %) in Senegal [36, 37]. Tanzanian women’s traditional roles and activities in the household are undergoing change, with increases in women’s status and power that are likely to promote reproductive health behaviors and service use [39–41]. In general Islamic traditions are believed to negatively influence women’s status, and women’s low social status is negatively related to maternal health services in Senegal [42, 43]. Yet Senegalese women have been renowned in their socioeconomic and political participation (e.g., local women’s organizations, governmental efforts including gender sensitive programs and decentralization) [44, 45]. These advantages and freedom of mobility may represent women’s higher empowerment status and can positively influence delivery care use.

### Data

This study used data from the 2010–11 Senegal and 2010 Tanzania Demographic and Health Surveys (DHS), nationally representative household surveys that collected data on population, health, and nutrition issues. The study sample consisted of all births reported by currently married women that occurred in the five years preceding each survey. The final female study sample included 7033 women and 10,668 births in Senegal, and 4445 women and 6748 births in Tanzania (weighted).

In the survey, the total number of women (both currently married and unmarried) who gave birth during this period was 8148 in Senegal and 5349 in Tanzania. Questions on household decision-making participation were asked to currently married women only, thus unmarried women were dropped from the analysis. Furthermore, a few women were dropped for missing data on the decision-making questions (*n* = 11 in TZ) and the perceptions of gender norms questions (*n* = 119 in SN and 82 in TZ). Among the births to the study female sample, some births were excluded due to missing data on delivery assistance (*n* = 4 in SN, 24 in TZ). Observations were weighted using individual and household weights to adjust for differences in the probability of selection and interview among cases in the sample. Given that this study is a secondary data analysis of public available data, the study was considered exempt from IRB approval by the UCLA Institutional Review Board.

#### Dependent variable

*SBA use* at childbirth was operationalized as the use of an SBA at childbirth(s) in the five years preceding the survey. The variable was recoded as binary, in accordance with the WHO definition of SBAs [2]. The SBAs included doctor or assistant medical officer, clinical officer, nurse or midwife; non-SBAs included MCH aide, village health worker, Traditional Birth Attendant, relative or friend, other, or no-one at the delivery.

#### Independent variables

*Women’s education* served as a proxy measure of women’s status in this analysis. The survey asked women to report on the highest level of school that she had attended. The variable was recoded as: no formal education; primary attended; and secondary or higher attended.

*Women’s Empowerment* is operationalized through four dimensions, as determined by exploratory and confirmatory factor analysis (see below): Household decision-making power, perceptions of gender norms against violence, perceptions of sex negotiation, and age at first marriage.

A. *Household decision-making power* was examined as a summative variable. The survey asked women about their participation in decisions regarding household matters (e.g., own health care, major household purchases, and visits to family or relatives). The variables were first recoded into binary to indicate whether the respondent participated in the decision, either alone or jointly with their husband, or not. A summative variable captured the number of decisions in which women participated (scored 0–3).
B. Two sets of questions in the DHS focused on perceived gender norms. The first domain, *perceptions of gender norms against violence,* asked about women’s acceptance of wife-beating by her husband under five situations – if she goes out without telling him, neglects the children, argues with him, refuses to have sex with him, or burns the food. Each of the variables was first recoded as binary (i.e., yes or no) then summed to create a scale capturing the number of situations in which women do NOT accept the violence (scored 0–5), with higher numbers indicating lower acceptance of gender violence and more progressive gender norms.
C. The second domain, *perceptions of gender norms for sex negotiation* asked about women’s perceived ability to negotiate sexual relations – if the respondent can refuse having sex or can ask her partner to use a condom. The variables were recoded to determine if the respondent can refuse/ask, or not (i.e., cannot refuse/ask, don’t know, not sure, or depends). A summative variable captured the number of situations in which women think that they can negotiate with their husband (scored 0–2).
D. *Age at first marriage* was also included based on the theoretical and empirical importance of this construct as a strategic life event and reflection of women’s empowerment [9, 17]. A continuous variable was created by MEASURE, based on calculation using the date of the first marriage or union (“living with a man as if married”) and the date of birth of the respondent.

#### Control variables

*Sociodemographic characteristics of women and households* included women’s age, parity, employment for payment, household wealth, marital and household relationship, the gender composition of children, and the place of residence. Women’s age at the time of delivery was included as a continuous variable based on preliminary analysis indicating a linear relationship with SBA use. Parity (i.e., the birth order of the children) was a categorical variable (e.g., first birth; second or third birth; fourth birth or more). Employment for payment was a binary measure defined as a woman who had been employed for cash or in-kind in the last 12 months, or not. Household wealth was examined using household asset data, such as ownership of consumer items and home attributes. Principal component analysis was conducted by MEASURE DHS to develop a ranking of household wealth according to the scores, and households were then divided into quintiles [37]. Marital relationship was assessed as categorical – monogamous union, polygamous as a first wife, or polygamous as a second wife or lower – to examine the potential differences by the type of marital relationship and wife order. Household relationship was assessed as binary – if the respondent was a household head or not. The gender composition of children was examined as a binary variable - if the respondent had at least one living son or not at the time of the delivery. This variable was included based on evidence of son preference in Africa, specifically that having at least one son has been valued for continuing the family lineage and kinship ties, as well as transfer of property due to inheritance laws [46]. Place of residence indicated if the respondent lived in an urban or rural area. These control variables were available in both countries. Other important variables (e.g., religion and ethnicity) were examined in separate models, but are not presented in the final models as they were not available in the Tanzania dataset.

*Perceived difficulty in accessing health care* was also included as a control variable, which assessed if the respondent perceives difficulty when seeking health care. The survey asked: “When you are sick and want to get medical advice or treatment, is each of the following a big problem or not?” The answers were collected for each aspect: getting permission to go; getting money needed for advice/treatment; the distance to the healthy facility; or not wanting to go alone. The variables were first recoded into binary variables to show if the respondent perceived a big problem or not (i.e., not a big problem or not a problem at all) for the four aspects separately, then a summative scale was created (scored 0–4), with higher scores indicating higher perceived difficulties.

### Analytic strategies

Data analysis was conducted in three main steps. First, descriptive analyses were conducted using SAS 9.3. Second, exploratory and confirmatory factor analyses were conducted using Mplus 7.3 to identify and confirm the underlying structure of the indicators of empowerment [47]. Third, sequential regression analyses were conducted in SAS. The simple (unadjusted) logistic regression was conducted first to examine the bivariate associations between SBA use and each of the explanatory variables. Next, the multivariate logistic regression was conducted that included all of the control variables found to be significant in the bivariate models. Last, the final multivariate logistic regression models added the measures of women’s empowerment, followed by the addition of interaction terms between each of the empowerment domains and education. The variance inflation factor assessed multicollinearity of variables in the model and was shown to be below cut-off point of 10.

All of the analyses were conducted accounting for individual weights, clusters (i.e., Primary Sampling Unit), and sample strata using the survey analysis commands. Given that the study examined births occurring to women nested in households, this analysis corrected the standard errors for clustering by woman and household using the Taylor Series linearization method [48]. Model fit was assessed though Likelihood Ratio (LR) chi-square test and Wald chi-square test.

## Results

The descriptive results of women in this study are shown in Table 1. Almost half of Tanzanian women used an SBA at the last birth (50.1 %), as compared to almost 2-thirds of Senegalese women (66.3 %). Tanzanian women had higher levels of education, monogamous unions, and were more likely to live in rural areas as compared to Senegalese women; however, the mean age at marriage/union was the same (Mean 18.3 years in SN and TZ).

**Table 1:** Characteristics of participating women who gave birth(s) in last five years (weighted *n* = 7033 in SN; *n* = 4445 in TZ), Senegal and Tanzania DHS 2010

Variables	Senegal			Tanzania		
	Freq	Weighted		Freq	Weighted	
		Mean or Proportion	SE		Mean or Proportion	SE
Outcome						
Skilled Birth Attendant (SBA) use at the last birth	4251	66.30	1.27	2233	50.95	1.51
Demographics and perceived accessibility of health care						
Education						
Formal education attendance (in years)		1.79	0.08		5.01	0.10
No formal education	5577	70.54	1.21	1082	24.42	1.22
Primary attended	1384	20.74	1.01	2771	68.93	1.18
Secondary or above attended	490	8.71	0.57	556	6.65	0.52
Age at childbirth		29.40	0.12		29.38	0.15
Household wealth quintile						
Poorest	2264	22.38	1.31	818	19.58	1.08
Poorer	1882	20.95	1.18	957	22.61	0.96
Middle	1534	19.19	1.13	905	21.47	0.92
Richer	1056	19.85	1.34	954	19.99	1.12
Richest	715	17.63	1.12	775	16.35	1.14
Employment for payment						
Employed (currently or last 12 months)	3386	46.04	1.12	1717	38.07	1.10
Parity (Total # of children ever born to women)		3.81	0.04		3.90	0.05
Marital relationships						
Monogamous union	4909	68.19	0.83	3394	78.87	0.53
Polygamous as 1st wife	991	12.73	0.44	434	8.97	0.53
Polygamous as 2nd or lower	1550	19.08	0.55	549	12.16	0.82
Household head	322	4.98	0.38	251	5.67	0.47
Place of residence						
Urban	2267	39.95	1.62	878	21.67	1.18
Rural	5184	60.05	1.62	3531	78.33	1.18
Having son(s)	5687	80.87	0.63	3618	81.38	0.67
Perceived difficulty in accessing health care (Mean, scored 0–4)		1.23	0.04		0.53	0.02
Women’s empowerment proxy measures						
Household decision-making power (scored 0–3)		0.92	0.03		1.43	0.02
Perceptions against violence (0–5)		2.80	0.05		3.16	0.04
Perceptions for sex negotiation (0–2)		0.60	0.02		1.38	0.02
Age at first marriage		18.29	0.10		18.28	0.06

Characteristics related to births were also assessed including all births that women delivered in the last five years (weighted birth *n* = 10,668 in SN; *n* = 6748 in TZ). The proportion of SBA use at the recent birth(s) was 64.6 % in SN; 47.5 % in TZ. The mean of birth order of each birth was 3.67 in SN; 3.75 in TZ. The proportion of births that took place when women had living son(s) was 60.2 % in SN; 62.3 % in TZ

Frequency missing with demographic characteristics = 32 (with marital relationships), and 17 (with perceived difficulty in accessing health care) in Tanzania. Missing = 1 (with marital relationships) in Senegal

Overall, Tanzanian women reported higher levels of women’s status and empowerment as compared to women in Senegal. On average, Tanzanian women participated in more household decisions and reported more cases in which gender violence was not justified, as compared to Senegalese women (0.92 in SN and 1.43 in TZ out of 3 household decisions; 2.80 in SN and 3.16 in TZ out of 5 score regarding gender violence). Similarly, Tanzanian women reported higher perceived levels of negotiation in their sexual relations as compared to Senegalese women (Mean 0.60 in SN and 1.38 in TZ out of 2).

Results from the factor analyses identified and confirmed three underlying factors from the ten indicators related to household decision-making and perceptions of gender norms – household decision-making power, perceptions of gender norms against violence, and perceptions for sex negotiation (Table 2) (Eigenvalues >1.0). Age at first marriage had very low loadings (e.g., less than 0.2) on all of the identified factors, suggesting that this was a separate dimension from the others. Moreover, the correlations between these identified three factors were low (<0.313 in SN; < 0.252 in TZ), suggesting that each of them were distinct and may have had disparate effects on SBA use.

**Table 2:** Factor analysis for indicators of empowerment (weighted *n* = 7033 in Senegal; 4445 in Tanzania), Senegal and Tanzania DHS 2010

Latent construct	Aspects that survey asked	Factor loadings	
		Senegal	Tanzania
Household decision-making	Decision on own health care	0.916	0.795
	Decision on major household purchases	0.869	0.865
	Decision on visits to family or relatives	0.851	0.939
Perceptions of gender norms against violence	Violence if going out without telling husband	0.917	0.890
	Violence if neglects the children	0.933	0.922
	Violence if argues with him	0.963	0.929
	Violence if refuses to have sex with him	0.911	0.883
	Violence if burnsthe food	0.822	0.863
Perceptions of gendernorms for sex negotiation	Perceived ability in refusing sex	0.803	0.844
	Perceived ability in asking condom use	0.771	0.693

Factor loadings from the three factor models are presented. All the loadings are significant at *p* < 0.05

Model fit statistics: [EFA for Senegal] RMSEA = 0.034, CFI = 0.996, TLI = 0.989, SRMS = 0.013

[EFA for Tanzania] RMSEA = 0.036, CFI = 0.996, TLI = 0.989, SRMS = 0.018

Tables 3 and 4 show the results of the sequential regression analyses predicting the odds of using an SBA at childbirth in Senegal and Tanzania (See model statistics in the tables. *p* < 0.05. P-values are also reported in the table). As shown in the bivariate model (Model 1 in Tables 3 and 4), most of the explanatory variables show statistically significant associations with SBA use in both settings, including women’s education, the main independent variable.

**Table 3:** Bivariate and multivariate logistic regression analyses of SBA use (weighted *n* = 10,668 in Senegal), Senegal DHS 2010

		Model 1 unadjusted (bivariate)				Model 2 adjusted				Model 3 final adjusted			
		OR	p	CI		OR	p	CI		OR	p	CI	
Independent variable	(Ref. = Primary edu)												
Women’s education	No education	0.355	<.001	0.303	0.415	0.888	0.199	0.741	1.064	0.972	0.758	0.809	1.167
	Secondary or above	2.064	<.001	1.457	2.922	0.994	0.978	0.659	1.501	0.937	0.759	0.616	1.423
Control variables													
Age at childbirth		1.003	0.439	0.995	1.011	1.029	<.001	1.018	1.041	1.017	0.012	1.004	1.031
Household wealth	(Ref. = Poorest)												
	Poorer	2.476	<.001	2.165	2.833	2.275	<.001	1.982	2.612	2.183	<.001	1.900	2.508
	Middle	6.927	<.001	5.927	8.097	4.547	<.001	3.84	5.384	4.273	<.001	3.604	5.067
	Richer	17.985	<.001	14.295	22.627	7.584	<.001	5.890	9.765	6.740	<.001	5.220	8.702
	Richest	52.422	<.001	36.208	75.896	18.721	<.001	12.88	27.22	15.978	<.001	10.944	23.327
Parity	(Ref. = 4th or more)												
	First birth	2.666	<.001	2.330	3.050	2.256	<.001	1.797	2.832	1.993	<.001	1.566	2.537
	Second or third	1.535	<.001	1.370	1.719	1.274	0.002	1.091	1.489	1.153	0.093	0.977	1.360
Employment	(Ref. = not employed)	1.095	0.115	0.978	1.225	0.788	<.001	0.694	0.894	0.797	<.001	0.703	0.904
Household head	(Ref. = not head)	1.693	<.001	1.261	2.274	1.166	0.367	0.835	1.627	1.154	0.409	0.821	1.624
Urban residence	(Ref. = rural)	10.066	<.001	8.594	11.790	3.032	<.001	2.526	3.640	2.854	<.001	2.377	3.426
Marital relationship	(Ref. = monogamous)												
	Polygamous as 1st wife	0.630	<.001	0.533	0.744	0.772	0.006	0.641	0.929	0.814	0.030	0.676	0.980
	2nd or lower	0.648	<.001	0.567	0.741	0.733	<.001	0.630	0.853	0.764	<.001	0.656	0.889
Having son(s)	(Ref. = no living son)	0.565	<.001	0.509	0.627	0.858	0.051	0.736	1.000	0.868	0.071	0.743	1.012
Perceived difficulty in accessing health care		0.655	<.001	0.625	0.687	0.864	<.001	0.825	0.905	0.865	<.001	0.825	0.907
	(scored 0–4)												
Women’s empowerment proxy measures													
Household decision-making power (0–3)		1.229	<.001	1.169	1.293					1.025	0.394	0.969	1.084
Perception against violence (0–5)		1.306	<.001	1.271	1.342					1.091	<.001	1.059	1.124
Perception for sex negotiation (0–2)		1.508	<.001	1.397	1.627					1.161	<.001	1.064	1.267
Age at first marriage		1.131	<.001	1.115	1.146					1.027	0.002	1.010	1.044
Intercept (coefficient)						−1.267	<.001			−1.704	<.001		
Model statistics													
LR (Chi-square)						3670.2785				3762.405			
Wald (Chi-square)						1303.6847				1325.9176			
DF						16				20			
p						<.001				<.001			

Model 1 (simple binary regression model) was assessed by each explanatory variable, and the model statistics of each model are not reported in the table. For the overall association, wald chi-square tests (from Type 3 Analysis of Effects) were assessed with education, wealth, parity, and marital relationship, showing significance at *p* < .001

**Table 4:** Bivariate and multivariate logistic regression analyses of SBA use for births (weighted *n* = 6748 in Tanzania), Tanzania DHS 2010

Variables		Model 1 unadjusted (bivariate)				Model 2 adjusted				Model 3 final adjusted			
		OR	p	CI		OR	p	CI		OR	p	CI	
Independent variable	(Ref.=Primary edu)												
Highest education	No education	0.457	<.001	0.386	0.542	0.667	<.001	0.546	0.814	0.702	<.001	0.574	0.858
	Secondary or above	5.564	<.001	4.088	7.573	1.515	0.009	1.111	2.066	1.428	0.024	1.047	1.946
Control variables													
Age at childbirth		0.986	0.007	0.976	0.996	1.049	<.001	1.032	1.067	1.040	<.001	1.021	1.060
Household wealth	(Ref.=Poorest)												
	Poorer	1.160	0.155	0.942	1.451	1.024	0.835	0.816	1.286	1.013	0.914	0.805	1.274
	Middle	1.844	<.001	1.487	2.286	1.531	<.001	1.217	1.925	1.528	<.001	1.214	1.923
	Richer	3.612	<.001	2.862	4.557	2.140	<.001	1.659	2.759	2.170	<.001	1.680	2.803
	Richest	21.612	<.001	15.681	29.787	6.72	<.001	4.033	9.141	5.836	<.001	3.895	8.744
Parity	(Ref.=4th or more)												
	First birth	2.757	<.001	2.315	3.283	3.134	<.001	2.297	4.274	2.936	<.001	2.120	4.066
	Second or third	1.731	<.001	1.502	1.996	1.901	<.001	1.537	2.350	1.778	<.001	1.422	2.223
Employment	(Ref.= not employed)	2.163	<.001	1.867	2.506	1.230	0.017	1.038	1.457	1.197	0.039	1.009	1.420
Household head	(Ref.= not head)	0.836	0.235	0.622	1.124	1.196	0.313	0.845	1.693	1.114	0.545	0.785	1.583
Urban residence	(Ref.=Rural)	7.305	<.001	5.617	9.499	2.182	<.001	1.582	3.011	2.183	<.001	1.589	2.999
Marital relationship	(Ref.=monogamous)												
	Polygamous as 1st wife	0.401	<.001	0.314	0.513	0.541	<.001	0.414	0.707	0.566	<.001	0.433	0.739
	2nd or lower	0.560	<.001	0.449	0.699	0.639	<.001	0.494	0.827	0.672	0.003	0.519	0.870
Having son(s)	(Ref.=No living son)	0.550	<.001	0.482	0.627	0.849	0.098	0.699	1.031	0.852	0.105	0.701	1.034
Perceived difficulty in accessing health care		0.607	<.001	0.561	0.657	0.732	<.001	0.672	0.798	0.739	<.001	0.678	0.805
	(scored 0–4)												
Women’s empowerment proxy measures													
Household decision-making power (0–3)		1.208	<.001	1.140	1.280					1.129	<.001	1.056	1.206
Perceptions against violence (0–5)		1.112	<.001	1.072	1.153					1.018	0.421	0.975	1.062
Perceptions for sex negotiation (0–2)		1.376	<.001	1.256	1.507					1.108	0.053	0.999	1.230
Age at first marriage		1.102	<.001	1.075	1.130					1.022	0.120	0.994	1.050
Intercept (coefficient)						−1.983	<.001			−2.477	<.001		
Model statistics													
LR (Chi-square)						1635.0332				1683.3702			
Wald (Chi-square)						751.1497				755.8300			
DF						16				20			
p						<.001				<.001			

Model 1 (simple binary regression model) was assessed by each explanatory variable, and the model statistics of each model are not reported in the table. For the overall association, wald chi-square tests (from Type 3 Analysis of Effects) were assessed with education, wealth, parity, and marital relationship, showing significance at *p* < .001

As displayed in Tables 3 and 4, the adjusted models (Model 2) indicated that women’s education was significantly and directly associated with SBA use in Tanzania, but not in Senegal. Births occurring to Tanzanian women with no education had 33.3 % lower odds of being attended by an SBA, and women with secondary or higher education had 51.5 % higher odds of being attended by an SBA, as compared to births to Tanzanian women with primary education.

The associations between sociodemographic characteristics and SBA use showed similarities across the two settings. Women’s age at delivery, household wealth, and urban residence were positively associated with SBA use, while polygamous union (either as first wife, or second or lower) and perceived difficulty in accessing health care were negatively associated with SBA use in both settings. Parity was also inversely related to SBA use such that women having their first birth were more likely to use an SBA (OR = 3.13 and 2.26 in TZ and SN, respectively), as compared to the fourth or higher order birth. Employment for payment was significantly associated with SBA use, but in the opposite directions in the two settings – positively in Tanzania (OR = 1.23), and negatively in Senegal (OR = 0.79).

In the final multivariate model (Model 3 in Tables 3 and 4), women’s education was significantly associated with SBA use in Tanzania even after controlling for the empowerment measures, but not in Senegal. The inclusion of the women’s empowerment variables appeared to diminish the effects of some of the demographic variables; however, most of the relationships were significant in the final, adjusted models (Model 3 in Table 3 and 4).

The association of SBA use with the women’s empowerment variables varied by proxy measure and by country. For example, household decision-making participation was the only measure that is significantly associated with SBA use in Tanzania, such that for every additional household decision in which women participated, they had 12.9 % higher odds of using SBA. This relationship, however, was not significant in Senegal. Conversely, perceptions against violence, perceptions for sex negotiation and age at first marriage were significantly and positively associated with SBA use in Senegal, but were not associated with SBA use in Tanzania. In Senegal, for each unit increase in gender norms against violence and sex negotiation, there were higher odds of SBA use (OR = 1.091 and OR = 1.161, respectively). Similarly, a 1-year increase in age at first marriage was related to 2.7 % higher odds of SBA use in Senegal.

Lastly, moderation analysis was conducted to assess if the effect of women’s empowerment on SBA use differs by women’s education –In Tanzania, there was an interaction between decision-making power and education, though these effects were at borderline significance (*p* = 0.07). Specifically, the magnitude of effect of decision-making on SBA use was larger among women with secondary or higher education relative to women with primary education (*p* < 0.05, OR = 1.271, CI = 1.006, 1.606), whereas there was no significant difference in this magnitude between no formal education and primary education. In Senegal, none of these interaction terms showed significance.

## Discussion

This study employed a multidimensional operationalization of women’s empowerment to examine the relationship of women’s status and empowerment with SBA use at childbirth in two distinct settings of sub-Saharan Africa – Senegal and Tanzania. The results confirmed that not only are the constructs of women’s status and empowerment multidimensional, but also that the influences of these constructs on SBA use vary across these two settings.

There are three key findings from this analysis. First, this study demonstrated the varied relationship of women’s formal education and SBA use by setting. Despite evidence generally demonstrating women’s education as a positive determinant of maternal and child health [5, 6] including substantial evidence from African studies [18–30], formal education was positively and directly related to SBA use in Tanzania, but not in Senegal. This finding may suggest that formal education may not always be the most appropriate proxy measure of women’s status in some settings. For example, in Senegal where non-formal and religious education is common and recognized (e.g., Islamic schools and/or education), it may be more appropriate to measure the additional forms of knowledge sharing and their potential benefits for women with respect to health care-seeking [49]. It may also be that given that the simple bivariate association was significant between formal education and SBA use in Senegal, the influence of education is likely to be attenuated by other important sociodemographic characteristics (e.g., household wealth) and/or by the inclusion of the women’s empowerment proxy measures. This highlights the importance of analyses that investigate both the direct and indirect pathways between women’s status and women’s empowerment, as well as potential moderating effects, as they related to the health and well-being of women and their families.

In fact, the moderation analysis indicated a synergetic effect between decision-making power and education in Tanzania, suggesting that improvement in *both* empowerment and education could have an accelerated impact on increasing SBA use. Although several African studies have examined the influence of education on delivery care use [18–30], only one known African study examined the moderation effects of education on the relationship between empowerment and maternal health service use [50]. On the other hand, there was no such evidence of a moderation effect in Senegal, highlighting varied influences of women’s status and empowerment on maternal health across settings. Findings regarding these varied influences underscores the importance of locally tailored maternal health interventions and programs that are culturally and contextually relevant [51].

Second, and related to the first finding, is that the relationship between individual dimensions of women’s empowerment and SBA use also varied across the two study settings. Women’s household decision-making power was significantly associated with SBA use only in Tanzania, while age at first marriage and perceptions of gender norms (against violence and for sex negotiation) were significantly associated with SBA use only in Senegal. These findings align with previous evidence and discussions that the notion of ‘women’s empowerment’ is contextually defined, and is likely comprised of different dimensions and domains across study settings [12, 52]. Evidence from this study is consistent with findings from other African studies that demonstrate varied relationships of the empowerment dimensions on maternal health service use [12, 14, 23, 28–30]. This evidence cautions against the replication of women’s empowerment programs across varied settings and populations without consideration of what constitutes empowerment in each context and how these programs could be best implemented to positively affect delivery care use.

Further evidence of these contextual differences was found in separate analyses indicating varied relationships between the women’s sociodemographic characteristics and the empowerment dimensions across settings (data not shown). For example, household wealth was positively associated with age at first marriage in Senegal, but not in Tanzania, suggesting that there may be different circumstances under which early marriage occurs, as well as differences in the potential implications of early marriage across these two settings. Further explication of these processes and pathways would be more feasible with longitudinal data and with the inclusion of other background characteristics for women, such as information on household characteristics of women’s natal families. Despite the importance of marriage and childbearing as “strategic life choices”, these events are often not considered as proxy measures of women’s empowerment in the existing literature. Future research efforts should consider expanded operationalizations of women’s empowerment to include these measures and, in alignment with recent global efforts, continue to explore both the predictors and potential consequences of early marriage and childbearing on subsequent health outcomes [53–57].

Third, the effect of women’s employment on SBA use also varied across the two settings, with employment being positively related to SBA use in Tanzania, and negatively related to SBA use in Senegal. Despite the fact that employment has been generally recognized as an enabling factor for women’s empowerment [9, 10], these mixed findings are consistent with recent research showing varied relationships between employment and delivery care use [23–26]. Findings from this and other studies suggest the various implications and reasons for women to work for payment – employment may represent women’s access to economic markets and their economic power in one context, while in another context, women may be more financially disadvantaged and may be forced to engage in earning activities irrespective of their choice and power [58]. Indeed these variations were also demonstrated in the separate regression analyses on women’s empowerment, finding that employment was differentially related to the empowerment measures across countries (data not shown). Together these findings call for further exploration of contextually-relevant measures of women’s economic power, as well as an examination of the extent to which these measures are associated with women’s “empowerment” and subsequent health outcomes [17].

This study entails some limitations despite its addressing several research gaps. This study employed cross-sectional survey datasets, thus any causal inference is tentative. Furthermore, due to the differences in survey sampling and weighting across the two contexts, it was not possible statistically test for differences between the two settings nor was it possible to directly compare coefficients across the two settings given concerns regarding unobserved heterogeneity in logistic regression [59]. Although the DHS surveys provide a set of tested and comparable measures across study settings, concerns have been raised regarding the extent to which these measures truly reflect women’s position and power within their respective societies, as well as the relevance or transferability of empowerment measures across study settings [12, 14].

Similarly, the DHS surveys only ask currently married women about household decision-making; thus, it is unknown if these findings are representative of unmarried women and adolescents. It is critical to examine girls’ empowerment and its effect on reproductive health service use and outcomes, especially in light of growing evidence that adolescents are at greater risk of delivery without skilled professionals, unsafe abortion, and maternal deaths [15, 53, 55, 57, 60, 61].

Last, it would have been ideal to examine additional, important variables, such as religion and ethnicity across the two settings, yet this information was only available for Senegal. However, a separate analysis of the Senegal data that included these variables produced similar conclusions (data not shown).

## Conclusions

Despite these limitations, this study is one of only a few studies that examined and incorporated a multidimensional investigation of women’s status and empowerment on delivery care use in sub-Saharan Africa. The study demonstrated the disparate influences of both sociodemographic characteristics, as well as women’s empowerment dimensions, on the use of an SBA in the two distinct settings. These findings highlight the important influence of women’s status and empowerment on SBA use, yet also underscore the importance of identifying potentially disparate influences across women’s empowerment dimensions, particularly when informing policies and programs that seek to promote SBA use for the reduction of maternal mortality.

## Abbreviations

DHS: Demographic and health surveys
SBA: Skilled birth attendant
